# A comparison of group prediction approaches in longitudinal discriminant analysis

**DOI:** 10.1002/bimj.201700013

**Published:** 2017-08-21

**Authors:** David M. Hughes, Riham El Saeiti, Marta García‐Fiñana

**Affiliations:** ^1^ Department of Biostatistics University of Liverpool Liverpool UK; ^2^ Department of Statistics University of Benghazi Benghazi Libya

**Keywords:** conditional distribution, longitudinal discriminant analysis, marginal distribution, multivariate, random‐effects distribution

## Abstract

Longitudinal discriminant analysis (LoDA) can be used to classify patients into prognostic groups based on their clinical history, which often involves longitudinal measurements of various clinically relevant markers. Patients' longitudinal data is first modelled using multivariate generalised linear mixed models, allowing markers of different types (e.g. continuous, binary, counts) to be modelled simultaneously. We describe three approaches to calculating a patient's posterior group membership probabilities which have been outlined in previous studies, based on the marginal distribution of the longitudinal markers, conditional distribution and distribution of the random effects. Here we compare the three approaches, first using data from the Mayo Primary Biliary Cirrhosis study and then by way of simulation study to explore in which situations each of the three approaches is expected to give the best prediction. We demonstrate situations in which the marginal or random‐effects approach perform well, but find that the conditional approach offers little extra information to the random‐effects and marginal approaches.

## 1 INTRODUCTION

Regular surveillance of a patient is a crucial step in determining if/when they will develop a particular disease. Patients thought to be at risk of a disease may be asked to attend periodic clinic appointments at which a number of clinically relevant variables (referred to as markers) are measured. These variables can be used to assess the risk that a particular patient has of developing a disease, possibly within a set time frame. Patients are classified into prognostic groups based on their risk of having the disease. One may consider a two‐group case where patients are allocated to the disease group or the no disease group. Alternatively, a multiple group scenario could be considered where patients are classified into groups based on the anticipated severity of their disease (e.g. stages of cancer). Such a clinical problem can be addressed using methods of discriminant analysis.

In many clinical settings, only the most recent information (obtained at the most recent clinic visit) is considered in assessing the risk of developing a disease for a particular patient. All previously gathered information is not considered, which could be an inefficient use of data. It may also be the case that the change in a patient's marker values over time is more informative in predicting their risk than simply the most recent value of the marker. To allow for a more flexible classification approach, in recent years, longitudinal discriminant analysis (LoDA) methods have been developed, which classify patients into prognostic groups based on their longitudinal history.

Methods of LoDA which consider only a single continuous marker have been developed by a number of authors (see, e.g. Brant et al., 2003; Kohlmann, Held, & Grunert, 2009; Lix & Sajobi, 2010; Tomasko, Helms, & Snapinn, 1999; Wernecke, Kalb, Schink, & Wegner, 2004). When multiple longitudinal markers have been collected at the follow‐up visits, it can be useful to include this additional information in the classification scheme. Extensions of LoDA for multiple continuous markers have been considered by Morrell, Brant, Sheng, and Metter (2012), Marshall, De la Cruz‐Mesía, Quintana, and Barón (2009), and Komárek, Hansen, Kuiper, van Buuren, and Lesaffre (2010). Fieuws, Verbeke, Maes, and Van Renterghem (2008) and Hughes, Komárek, Czanner, and Garcia‐Fiñana (2016) develop multivariate LoDA methods which can be used when the markers are not all continuous (e.g. counts or binary data).

In each of the LoDA methods referenced above, a linear mixed model is first used to model the longitudinal evolution of each marker for patients of known prognosis. A useful feature of mixed models is that random effects are used to allow patient‐specific deviation from the mean profile as well as to model correlation between observations of a marker at different time points and also between markers. In the case of markers of different types (Fieuws et al., 2008; Hughes et al., 2016), the linear mixed model is extended to a multivariate generalised linear mixed model (MGLMM). A MGLMM is fit separately to data from each prognostic group. The output of these models is then used in the LoDA to inform a classification rule. In other words, we use the longitudinal data on markers, from patients of known prognosis, to derive a classification rule which predicts the future disease status of a patient of unknown prognosis based on their individual longitudinal history.

Morrell, Brant, and Sheng (2007) specify three alternative ways to use the output from the mixed model to predict disease status, namely marginal, conditional and random‐effects prediction. In each case, the prediction has a different focus. For marginal prediction, the marginal distribution of the new patient's observed longitudinal data is used to predict their future status. That is, the prediction is focused on the mean evolution of the markers over time. We are interested in which of the group‐specific mean longitudinal profiles, calculated using the MGLMM, the new patient's trajectory is closest to. The conditional prediction replaces the marginal distribution with the conditional density of the observed longitudinal data given the estimate of the new patient's random effects. In this case, the prediction is based on the patient‐specific evolution of markers over time, ignoring any error in the variability of the patient's estimated random effects. This method could be thought of as comparing the mean longitudinal profiles for a subset of patients with ‘similar’ random effects in each group to the conditional longitudinal profile of the new patient. Finally, for random‐effects prediction the density of the patient's estimated random effects is used for prediction and the focus is on the patient‐specific evolution of the markers.

Most applications of LoDA have focused on the so called ‘marginal’ prediction approach. To the best of our knowledge, Morrell et al. (2007) were the first to propose the use of conditional and random‐effects prediction as alternatives. Relatively little work has been done to assess which of the three methods is most appropriate to use, or whether different approaches suit some scenarios more than others. In work that aims to identify patients with prostate cancer based on the evolution over time of prostate‐specific antigen (PSA), Morrell et al. (2007) and Morrell, Sheng, and Brant (2011) compare the three prediction approaches using a number of measures. In terms of sensitivity (proportion of correctly identified cancer cases) and lead time (mean time before clinical diagnosis that a patient is correctly predicted as a cancer case), the marginal method performs the best, whilst in terms of specificity (proportion of correctly identified non‐cases) and probability of correct classification (PCC), the random‐effects method performed the best. By contrast, Komárek et al. (2010) used the three methods to identify patients with primary biliary cirrhosis (PBC) based on three continuous longitudinal markers and concluded that the random‐effects method gave the best prediction. Hughes et al. (2016) also compared the three approaches to identify patients with refractory epilepsy and show that, in their application the marginal and conditional approaches performed similarly, with a slight preference for the marginal approach whilst the random‐effects approach performed poorly.

All previous comparisons of the three approaches to LoDA have been based on specific data sets and have provided different conclusions as to which approach works best. In this paper we investigate this matter further, by way of simulation study, to determine whether the three approaches are sensitive to different types of differences between the prognostic groups.

An outline of this paper is as follows. In Section 2, we give an overview of the LoDA methodology and explain in more detail the marginal, conditional and random‐effects approaches. Section 3 gives a real data application of LoDA using the PBC data available within the mixAK (Komárek & Komárková, 2014) package in R (R Core Team, 2016). We describe a simulation study comparing the three approaches in two different scenarios in Section 4. We highlight some conclusions in Section 5.

## 2 OVERVIEW OF LONGITUDINAL DISCRIMINANT ANALYSIS

### 2.1 Multivariate generalised linear mixed model

Our aim in this paper is to use data from patients of known prognosis to predict the group membership at some future point for new patients. We first introduce some notation following the definitions of Hughes et al. (2016). Each patient may belong to one of *G* groups based on a diagnosis at specific time *T*. We represent this by a value of the random variable $U\in \left\{0,\;\ldots ,\;G-1\right\}$, which is only observed at time *T*. We assume that for each patient, measurements are made on R≥1 markers at times $t_{r}=\left(t_{r,1},\;\ldots ,\;t_{r,n_{r}}\right)$, $t_{r,1}<\cdots <t_{r,n_{r}}<T$, r=1,…,R. In common with the entire MGLMM methodology, this approach does not require that each marker is measured at the same time points, or even the same number of times. Neither is it necessary for all patients to have the same number of measurements or identical visit schedules. For each marker, these longitudinal observations for a particular patient are denoted $Y_{r}=\left(Y_{r,1},\;\ldots ,\;Y_{r,n_{r}}\right),r=1,\ldots ,R$. The longitudinal evolution of each marker may depend on additional covariate vectors $v_{r,1},\;\ldots ,\;v_{r,n_{r}}\in R^{p_{r}}$ which we denote as C. We aim to use the information collected for a patient up until some t<T to predict the future group, *U*, to which the patient belongs. The prediction is based on the information gathered about the patient at time *t* and also all previous data for that patient.

We first fit separate MGLMMs to the longitudinal data for each prognostic group. The expected value (transformed by an appropriate link function) for the *j*‐th observation ($j=1,\;\ldots ,\;n_{r}$) of the *r*‐th marker (r=1,…,R) of a patient in group *g* (denoted $Y_{r,j}$) is given by

(1) $$h_{r}^{-1}\left\{E\left(Y_{r,j}\;|\;b,\;U=g\right)\right\}=x_{r,j}^{g⊤}\alpha_{r}^{g}+z_{r,j}^{g⊤}b_{r},\;r=1,\ldots ,R,\;j=1,\ldots ,n_{r},$$

where $h_{r}^{-1}$ is a chosen link function (chosen dependent on the particular exponential family distribution being modelled (e.g. normal, Poisson, Bernoulli), with possible dispersion parameters $\phi_{r}^{g}$), $x_{r,j}^{g}=x_{r,j}^{g}\left(C\right)$ and $z_{r,j}^{g}=z_{r,j}^{g}\left(C\right)$ are covariate vectors used in a model for the prognostic group *g* and $\alpha_{r}^{g}$, r=1,…,R, g=0,…,G−1 denotes unknown regression coefficients.

The unobserved random‐effects vector $b=\left(b_{1},\;\ldots ,\;b_{R}\right)$ accounts for possible correlation between repeated observations of the same marker and also different markers on the same patient. Typically, the random‐effects vector is assumed to jointly follow a normal distribution. However, Hughes et al. (2016) allow additional flexibility by specifying a mixture of normal distributions for the joint distribution of the random‐effects vector in each prognostic group (see also Komárek et al., 2010; Verbeke & Lesaffre, 1996). That is, they assume $b\;|\;U=g\;∼\;\sum_{k=1}^{K}w_{k}^{g}\;\mathrm{MVN}\left(\mu_{k}^{g},\;D_{k}^{g}\right)$, where MVN(μ,D) stands for a multivariate normal distribution with the mean vector ***μ*** and a covariance matrix D. The mixture distributions are weighted by a factor $w_{k},\left(k=1,\;\ldots ,\;K\right)$. This multivariate normal distribution has a density denoted as φ(·;μ,D).

To fit this MGLMM, we need to estimate fixed effects regression coefficients from (1), denoted $\psi^{g}:=\left(\alpha_{1}^{g},\;\ldots ,\;\alpha_{R}^{g},\;\phi_{1}^{g},\;\ldots ,\;\phi_{R}^{g}\right)$ and additionally mixture related parameters denoted $\theta^{g}:=\left(w^{g},\;\mu_{1}^{g},\;\ldots ,\;\mu_{K^{g}}^{g},\;D_{1}^{g},\;\ldots ,\;D_{K^{g}}^{g}\right)$. Full details of this MGLMM, which is based on the MGLMM proposed by Komárek and Komárková (2013) can be found in Hughes et al. (2016).

### 2.2 Group probabilities for individual patients

The aim of the discriminant analysis is to use the model parameters, $\psi^{g}$ and $\theta^{g}$, estimated from the MGLMM in each group, to classify a new patient based on their longitudinal history. An application of Bayes theorem gives the probability that a patient belongs to group *g* given their longitudinal and covariate data and the model parameters from the MGLMMs fit to patients of known status.

(2) $$P_{g,new}=\frac{\pi_{g}\;{\hat{f}}_{g,new}}{\sum_{\tilde{g}=0}^{G-1}\pi_{\tilde{g}}\;{\hat{f}}_{\tilde{g},new}}\;g=0,\;\ldots ,\;G-1,$$

where $\hat{f}$ denotes a predictive density of the observed markers given the group and model parameters (or in the case of the random‐effects approach, the density of the random effects given the group‐specific mixture parameters). Here the prior probabilities of belonging to each group are denoted by $\pi_{g}=P\left(U=g\right)$, g=0,…,G−1 and are often taken to be the proportions of the prognostic groups in the study population. In a frequentist setting, $f_{g,new}$ is estimated using the maximum likelihood estimates of the relevant model parameters in group *g*. The proposed MGLMM produces a likelihood function involving intractable integrals, and so instead Hughes et al. (2016) propose the use of Bayesian estimates of the group membership probabilities. In a Bayesian setting, $f_{g,new}$ is estimated as the mean of the posterior predictive density estimated from *M* samples from a Markov Chain Monte Carlo (MCMC) scheme (see Komárek & Komárková, 2013 for details of the MCMC procedure and also for the full specification of the Bayesian model). As already indicated, in this paper we investigate three different ways of specifying the predictive density $f_{g,new}$ in order to classify patients into prognostic groups.

### 2.3 Marginal prediction

The marginal prediction approach is the most commonly used approach in the LoDA literature. The aim of this approach is to compare the longitudinal profiles of a new patient to the group‐specific average profiles (computed from the historical data). The new patient is assigned to the prognostic group to which their longitudinal profiles lie closest. Here the predictive density $f_{g,new}$ is taken as the marginal density of $Y_{new}=\left(y_{new,1},\;\ldots ,\;y_{new,R}\right)$. That is,

$$P_{new,g}^{marg}\left(\psi ,\;\theta \right)=\frac{\pi_{g}\;f_{g,new}^{marg}\left(y_{1},\;\ldots ,\;y_{R};\;\psi^{g},\;\theta^{g},\;C\right)}{\sum_{\tilde{g}=0}^{G-1}\pi_{\tilde{g}}\;f_{\tilde{g},new}^{marg}\left(y_{1},\;\ldots ,\;y_{R};\;\psi^{\tilde{g}},\;\theta^{\tilde{g}},\;C\right)}\;g=0,\;\ldots ,\;G-1,$$

where $f_{g,new}^{marg}$ is the marginal density

(3) $$f_{g}^{marg}\left(y_{1},\;\ldots ,\;y_{R};\;\psi^{g},\;\theta^{g},\;C\right)=\int f_{g}^{cond}\left(y_{1},\;\ldots ,\;y_{R}\;|\;b;\;\psi^{g},\;C\right)\;f_{g}^{ranef}\left(b;\;\theta^{g}\right)\;db,$$

and $f_{g}^{cond}$ denotes a (conditional) density of the observed markers in the prognostic group *g* given the random‐effect vectors,

$$f_{g}^{cond}\left(y_{1},\;\ldots ,\;y_{R}\;|\;b;\;\psi^{g},\;C\right)=\prod_{r=1}^{R}\;\prod_{j=1}^{n_{r}}\;p_{r}\left(y_{r,j}\;|\;b;\;\psi^{g},\;C\right).$$

Here $p_{r}\left(\cdot \;|\;b;\;\psi^{g},\;C\right)$ denotes an exponential family density of the random variable $Y_{r,j}$ related to the GLMM (1). The random‐effects density, $f_{g}^{ranef}$ in (3), in the prognostic group *g* is

$$f_{g}^{ranef}\left(b;\;\theta^{g}\right)=\sum_{k=1}^{K^{g}}w_{k}^{g}\;\varphi (b;\;\mu_{k}^{g},\;D_{k}^{g}).$$

The group membership probabilities, $P_{g}\left(\psi ,\;\theta \right)$, are evaluated at each draw of the MCMC procedure and approximate group membership probabilities are calculated as the average across all *M* samples.

$${\hat{P}}_{new,g}^{marg}\;=\;\frac{1}{M}\sum_{m=1}^{M}\;P_{new,g}^{marg}\left(\psi^{\left(m\right)},\;\theta^{\left(m\right)}\right),\;g=0,\;\ldots ,\;G-1,$$

### 2.4 Conditional prediction

For the conditional approach, the marginal distribution of $Y_{new}$ is replaced by the conditional distribution of $Y_{new}$ given a patient‐specific estimate of the unknown random effects as the form of the predictive density $f_{g,new}$. That is, we use $f_{g}^{cond}$ in place of $f_{g,new}$ in (2) and the conditional group membership probabilities are calculated as the average over *M* draws in the MCMC procedure

$${\hat{P}}_{new,g}^{cond}=\frac{1}{M}\;\sum_{m=1}^{M}\;\frac{\pi_{g}\;f_{g}^{cond}(y_{new,1},\;\ldots ,\;y_{new,R}\;|\;b_{new}^{g,(m)};\;\psi^{g,(m)})}{\sum_{\tilde{g}=0}^{G-1}\pi_{\tilde{g}}\;f_{\tilde{g}}^{cond}(y_{new,1},\;\ldots ,\;y_{new,R}\;|\;b_{new}^{\tilde{g},\left(m\right)};\;\psi^{\tilde{g},\left(m\right)})},\;g=0,\;\ldots ,\;G-1.$$

In this case, the random effects for the patient must be estimated, and the mean of the conditional distribution of the random effects given the patient data and the model parameters is typically used (Hughes et al., 2016; Komárek et al., 2010).

### 2.5 Random‐effects prediction

Random‐effects prediction focuses on the patient‐specific evolution of the longitudinal markers. As with the conditional approach, a suitable estimate of the patient‐specific random effect is required. The predictive density $f_{g,new}$ is taken to be the density of the random effects evaluated at the patient and group‐specific estimate of the random effect given the marker data, $f_{g}^{ranef}$. As previously, the mean group membership probabilities, to be used for classification, are calculated by averaging over the MCMC samples.

$${\hat{P}}_{new,g}^{ranef}=\frac{1}{M}\;\sum_{m=1}^{M}\;\frac{\pi_{g}\;f_{g}^{ranef}(b_{new}^{g,(m)};\;\theta^{g,(m)})}{\sum_{\tilde{g}=0}^{G-1}\pi_{\tilde{g}}\;f_{\tilde{g}}^{ranef}(b_{new}^{\tilde{g},\left(m\right)};\;\theta^{\tilde{g},\left(m\right)})},\;g=0,\;\ldots ,\;G-1.$$

### 2.6 Classification rules

The estimates of the marginal, conditional and random‐effects group membership probabilities for a patient, are then used to classify the patient into a prognostic group. Typically, for each scheme, the patient is assigned to the group with the largest probability. For example, for marginal prediction of the future status of a (new) patient would be ${\mathrm{argmax}}_{g=0,\;\ldots ,\;G-1}{\hat{P}}_{new,g}^{marg}$. This is equivalent to setting a cut‐off probability of 0.5 in the two‐group classification case. An alternative scheme would be to classify a patient into a group only if the probability of belonging to that group is greater than a chosen cut‐off *c*. This cut‐off is typically chosen through analysis of a receiver operating characteristic (ROC) curve (by selecting for example the cut‐off that gives the closest point on the ROC curve to the top left corner). In the Bayesian methods outlined, the MGLMMs do not need to be refitted to classify new patients. Simply the group membership probabilities are calculated and an appropriate classification rule is applied. In this paper, all the longitudinal information gathered on a patient up until the time of prediction is used to calculate a patient's group membership probabilities. However, the LoDA approach can also be used to calculate dynamic predictions where the patient's group probabilities are recalculated each time new information becomes available, as was described in Hughes et al. (2016).

## 3 PRIMARY BILIARY CIRRHOSIS DATA

Komárek et al. (2010) present an application of LoDA to data from the Dutch Multicenter Primary Biliary Cirrhosis study, using three continuous markers to show that, for this application, the random‐effects prediction approach performs better than the marginal and conditional approaches. A similar PBC data set (The Mayo clinic trial Dickson, Grambsch, Fleming, Fisher, & Langworthy, 1989; Murtaugh et al., 1994) is presented in Komárek and Komárková (2013) in the context of cluster analysis and this data set is included within the mixAK (Komárek & Komárková, 2014) package in R (R Core Team, 2016; the data are available in Appendix D of Fleming & Harrington, 1991, and also electronically at http://lib.stat.cmu.edu/datasets/pbcseq). We present here an application of multivariate LoDA using continuous, binary and Poisson markers to the Mayo PBC data. PBC is a rare, but fatal liver disease. The initial study aimed to determine if the use of D‐penicillamine increased the length of patient survival. Data on a large number of clinical parameters were recorded for 312 patients over a median of 6.3 years per patient.

**Figure 1:**
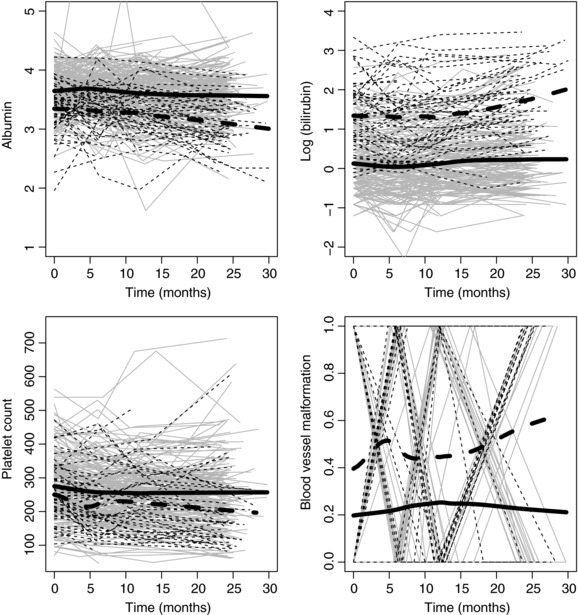
Observed longitudinal profiles of albumin (mg/dl), log(bilirubin) (log(mg/dl)), platelet counts and blood vessel malformation for patients who are known to be alive at 5 years (Group 0, solid lines) and who die between 2.5 and 5 years (Group 1, dashed lines); the thick lines show fitted mean over time

Our aim is to use only the data collected up until 2.5 years to predict those patients who will die or require transplant within five years. Therefore, we focus on patients known to be alive and without a liver transplant after two and a half years, and for whom we also know their condition after five years. We identified 202 patients who were known to be alive without transplant after five years and 51 patients who died or had a liver transplant at some point in time between 2.5 and 5 years. Four longitudinal markers were considered for the multivariate LoDA, specifically the continuous markers albumin and logarithmic serum bilirubin, the platelet count (Poisson) and a binary marker indicating blood vessel malformations. See Figure 1 for individual patient profiles for each marker.

The GLMM for each of the continuous and count markers contained a random intercept and a random time slope, whilst the GLMM for the binary marker contained a random intercept and a fixed effect for time (in each model time was recorded in months). To keep things simple, and to allow easy comparison with the simulations presented in Section 4 we consider a one‐component mixture distribution (i.e. K=1, see Hughes et al., 2016) for the random‐effects distribution.

**Figure 2:**
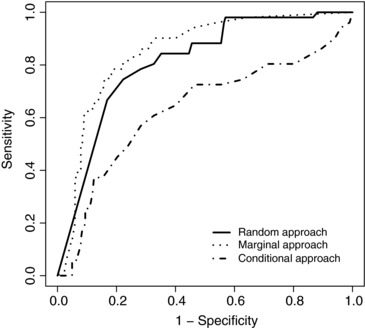
Receiver‐operating characteristic curves of the LoDA using the random‐effects (solid), marginal (dotted) and conditional (dot‐dashed) prediction methods for PBC data

To predict the group membership of a patient, separate MGLMMs were fit to patients in each group excluding the data of the patient for whom prediction was being made. Table 1 shows the predictive accuracy of this leave‐one‐out cross‐validation study applied to the PBC data. The cut‐off was chosen to give the point closest to the top left corner of the ROC curve (Fig. 2) and the predictive accuracies relate to the cut‐off reported for each of the three methods. For the PBC data, all three methods give reasonably good prediction of whether or not a patient will be alive without transplant after five years of observation. However, the conditional approach gives worse predictions than the other two approaches, whilst the marginal approach gives the best prediction, with 78% of patients who will die or require transplant correctly identified (Sensitivity), 81% of patients who will be alive without transplant correctly identified (Specificity) and 81% of patients correctly identified overall. The area under ROC curve (AUC) summarises the performance of the classification methods over a range of cut‐offs and again the marginal prediction approach performs best. A positive predictive value (PPV) of 51% for the marginal approach shows the percentage of patients predicted to die or require transplant who ultimately did die or require transplant, whilst the negative predictive value (NPV) of 94% shows that 94% of patients predicted to be alive without transplant were indeed alive after five years without requiring transplant.

**Table 1:** Prediction accuracy from leave‐one‐out cross‐validation of random‐effects, marginal and conditional prediction for PBC data

	Random	Marginal	Conditional
Cut‐off	0.98	0.21	0.12
Sensitivity	0.75	0.78	0.61
Specificity	0.78	0.81	0.67
PCC	0.77	0.81	0.66
AUC	0.81	0.85	0.63
PPV	0.46	0.51	0.32
NPV	0.92	0.94	0.94

Profiles of the longitudinal markers in each group are shown in Figure 1. The thick lines represent the group average profile. Except for the platelet count, the mean group profiles clearly differ between the two groups (i.e. there exist marginal differences between the groups). The variability around the mean group profiles also appears to be different between the groups. These factors explain why the marginal and random‐effects approaches give good classification accuracy. Komárek et al. (2010) find that the random‐effects approach gives best prediction when using LoDA on the Dutch Multicenter Primary Biliary Cirrhosis data. However, they have approximately 10 years of follow‐up per patient with 13 observations per patient on average (every three months for the first year and then annually after that). We believe that the increased number of observations per patient allowed better estimation of the patient‐specific random effects, hence showing the improved prediction accuracy from the random‐effects approach. In fact, when we analysed all the Mayo PBC data (average of 7.03 visits per patient), and not just the first 2.5 years of data per patient (3.53 visits per patient) we also observed that the random‐effects approach gave better predictive accuracy. This suggests that the random‐effects approach can give added information and improvement in classification accuracy, but only if the random effects are precisely estimated.

## 4 SIMULATION STUDY

In Section 3, we presented an application of multivariate LoDA in which the marginal and the random‐effects prediction method gave good predictive accuracy. However, as noted in Section 1, there have been contrasting findings in published studies as to which prediction method is best (Hughes et al., 2016; Morrell et al., 2007, 2011). This suggests that the type of data being considered influences which method will give the best prediction accuracy.

To explore this further, we considered simulation scenarios, based on the PBC data, but altered to reflect situations in which we believed the marginal and conditional approaches would lead to the most accurate predictions. We simulate data from 200 patients who are alive after five years without requiring transplant and 50 patients who were alive at 2.5 years but subsequently died or required transplant before five years, approximately reflecting the prevalence of the PBC data. For each patient, we simulated four clinic visits (following Komárek & Komárková, 2013). The first visit occurred at t=0 and the remaining visits were generated from uniform distributions in the intervals (170, 200), (350, 390) and (710, 770) days. This approximates to a visit after six months and then visits at one and two years. To more easily control the simulation differences we consider only a single normal distribution for the random effects (i.e. K=1, no mixture).

In each group, marker values were simulated from the appropriate GLMM at each of the four time points for each of the four markers considered in Section 3 (albumin, log(bilirubin), platelet count and blood vessel malformations). The values used to simulate the marker data from a GLMM are given in Table 2. We consider two alternative scenarios in our simulations.

**Table 2:** Parameter estimates for the PBC data and the modifications used for each simulation scenario

	Group 0			Group 1		
	PBC Data	Scenario 1	Scenario 2	PBC Data	Scenario 1	Scenario 2
**Albumin**						
E[Albumin:Intercept]	3.69	3.69	3.00	3.39	3.39	3.00
E[Albumin:slope]	−6.83$\;\times 10^{-3}$	−6.83$\;\times 10^{-3}$	0.00	−1.44$\;\times 10^{-2}$	−1.44$\;\times 10^{-2}$	0.00
SD[Albumin:Intercept]	2.73$\;\times 10^{-1}$	2.64$\;\times 10^{-1}$	6.50$\;\times 10^{-2}$	2.64$\;\times 10^{-1}$	2.64$\;\times 10^{-1}$	6.50$\;\times 10^{-2}$
Corr[Albumin:Intercept,Albumin:slope]	−8.60$\;\times 10^{-2}$	−6.46$\;\times 10^{-2}$	−6.46$\;\times 10^{-2}$	−6.46$\;\times 10^{-2}$	−6.46$\;\times 10^{-2}$	−6.46$\;\times 10^{-2}$
Corr[Albumin:Intercept,log(Bilirubin):Intercept]	−2.48$\;\times 10^{-1}$	−1.97$\;\times 10^{-1}$	−1.97$\;\times 10^{-1}$	−1.97$\;\times 10^{-1}$	−1.97$\;\times 10^{-1}$	−1.97$\;\times 10^{-1}$
Corr[Albumin:Intercept,log(Bilirubin):slope]	−1.10$\;\times 10^{-1}$	2.11$\;\times 10^{-1}$	2.11$\;\times 10^{-1}$	2.11$\;\times 10^{-1}$	2.11$\;\times 10^{-1}$	2.11$\;\times 10^{-1}$
Corr[Albumin:Intercept,Platelet:Intercept]	1.82$\;\times 10^{-1}$	1.91$\;\times 10^{-1}$		1.91$\;\times 10^{-1}$	1.91$\;\times 10^{-1}$	
Corr[Albumin:Intercept,Platelet:slope]	5.72$\;\times 10^{-2}$	1.09$\;\times 10^{-1}$		1.09$\;\times 10^{-1}$	1.09$\;\times 10^{-1}$	
Corr[Albumin:Intercept,Blood vessel malformation:Intercept]	−2.27$\;\times 10^{-1}$	−3.48$\;\times 10^{-1}$		−3.48$\;\times 10^{-1}$	−3.48$\;\times 10^{-1}$	
SD[Albumin:slope]	4.30$\;\times 10^{-3}$	7.76$\;\times 10^{-3}$	7.76$\;\times 10^{-3}$	7.76$\;\times 10^{-3}$	7.76$\;\times 10^{-3}$	7.76$\;\times 10^{-3}$
Corr[Albumin:slope,log(Bilirubin):Intercept]	−2.91$\;\times 10^{-1}$	1.57$\;\times 10^{-3}$	1.57$\;\times 10^{-3}$	1.57$\;\times 10^{-3}$	1.57$\;\times 10^{-3}$	1.57$\;\times 10^{-3}$
Corr[Albumin:slope,log(Bilirubin):slope]	−6.50$\;\times 10^{-1}$	−2.33$\;\times 10^{-1}$	−2.33$\;\times 10^{-1}$	−2.33$\;\times 10^{-1}$	−2.33$\;\times 10^{-1}$	−2.33$\;\times 10^{-1}$
Corr[Albumin:slope,Platelet:Intercept]	8.89$\;\times 10^{-2}$	−2.57$\;\times 10^{-1}$		−2.57$\;\times 10^{-1}$	−2.57$\;\times 10^{-1}$	
Corr[Albumin:slope,log(Bilirubin):slope]	2.96$\;\times 10^{-1}$	−2.60$\;\times 10^{-1}$		−2.60$\;\times 10^{-1}$	−2.60$\;\times 10^{-1}$	
Corr[Albumin:slope,Blood vessel malformation:Intercept]	−2.93$\;\times 10^{-1}$	2.27$\;\times 10^{-1}$		2.27$\;\times 10^{-1}$	2.27$\;\times 10^{-1}$	
SD[Albumin:residual]	3.18$\;\times 10^{-1}$	3.18$\;\times 10^{-1}$	3.14$\;\times 10^{-1}$	3.14$\;\times 10^{-1}$	3.14$\;\times 10^{-1}$	1.59$\;\times 10^{-1}$
**log(Bilirubin)**						
E[log(Bilirubin):Intercept]	2.13$\;\times 10^{-2}$	2.13$\;\times 10^{-2}$	1.00	1.23	1.23	1.00
E[log(Bilirubin):slope]	9.94$\;\times 10^{-3}$	9.94$\;\times 10^{-3}$	0.00	2.38$\;\times 10^{-2}$	2.38$\;\times 10^{-2}$	0.00
SD[log(Bilirubin):Intercept]	6.88$\;\times 10^{-1}$	8.45$\;\times 10^{-1}$	1.12$\;\times 10^{-2}$	8.45$\;\times 10^{-1}$	8.45$\;\times 10^{-1}$	1.12$\;\times 10^{-2}$
Corr[log(Bilirubin):Intercept,log(Bilirubin):slope]	2.32$\;\times 10^{-1}$	−1.75$\;\times 10^{-1}$	−1.75$\;\times 10^{-1}$	−1.75$\;\times 10^{-1}$	−1.75$\;\times 10^{-1}$	−1.75$\;\times 10^{-1}$
Corr[log(Bilirubin):Intercept,Platelet:Intercept]	−1.66$\;\times 10^{-1}$	2.47$\;\times 10^{-1}$		2.47$\;\times 10^{-1}$	2.47$\;\times 10^{-1}$	
Corr[log(Bilirubin):Intercept,Platelet:slope]	−2.04$\;\times 10^{-1}$	−1.87$\;\times 10^{-1}$		−1.87$\;\times 10^{-1}$	−1.87$\;\times 10^{-1}$	
Corr[log(Bilirubin):Intercept,Blood vessel malformation:Intercept]	3.42$\;\times 10^{-1}$	2.70$\;\times 10^{-1}$		2.70$\;\times 10^{-1}$	2.70$\;\times 10^{-1}$	
SD[log(Bilirubin):slope]	1.12$\;\times 10^{-2}$	1.49$\;\times 10^{-2}$	1.49$\;\times 10^{-2}$	1.49$\;\times 10^{-2}$	1.49$\;\times 10^{-2}$	1.49$\;\times 10^{-2}$
Corr[log(Bilirubin):slope,Platelet:Intercept]	1.44$\;\times 10^{-2}$	−1.69$\;\times 10^{-1}$		−1.69$\;\times 10^{-1}$	−1.69$\;\times 10^{-1}$	
Corr[log(Bilirubin):slope,Platelet:slope]	−2.40$\;\times 10^{-1}$	1.25$\;\times 10^{-1}$		1.25$\;\times 10^{-1}$	1.25$\;\times 10^{-1}$	
Corr[log(Bilirubin):slope,Blood vessel malformation:Intercept]	3.05$\;\times 10^{-1}$	8.13$\;\times 10^{-3}$		8.13$\;\times 10^{-3}$	8.13$\;\times 10^{-3}$	
SD[log(Bilirubin):residual]	3.38$\;\times 10^{-1}$	3.38$\;\times 10^{-1}$	3.95$\;\times 10^{-1}$	3.96$\;\times 10^{-1}$	3.96$\;\times 10^{-1}$	1.69$\;\times 10^{-1}$
**Platelet count**						
E[Platelet:Intercept]	5.54	5.54		5.46	5.46	
E[Platelet:slope]	−4.29$\;\times 10^{-3}$	−4.29$\;\times 10^{-3}$		−1.14$\;\times 10^{-2}$	−1.14$\;\times 10^{-2}$	
SD[Platelet:Intercept]	3.73$\;\times 10^{-1}$	3.45$\;\times 10^{-1}$		3.45$\;\times 10^{-1}$	3.45$\;\times 10^{-1}$	
Corr[Platelet:Intercept,Platelet:slope]	−4.64$\;\times 10^{-2}$	6.14$\;\times 10^{-2}$		6.14$\;\times 10^{-2}$	6.14$\;\times 10^{-2}$	
Corr[Platelet:Intercept,Blood vessel malformation:Intercept]	−7.41$\;\times 10^{-2}$	−2.48$\;\times 10^{-1}$		−2.48$\;\times 10^{-1}$	−2.48$\;\times 10^{-1}$	
SD[Platelet:slope]	5.66$\;\times 10^{-3}$	1.51$\;\times 10^{-2}$		1.51$\;\times 10^{-2}$	1.51$\;\times 10^{-2}$	
Corr[Platelet:slope,Blood vessel malformation:Intercept]	−1.68$\;\times 10^{-1}$	−8.03$\;\times 10^{-2}$		−8.03$\;\times 10^{-2}$	−8.03$\;\times 10^{-2}$	
**Blood vessel malformations**						
E[Blood vessel malformation:Intercept]	−2.54	−2.54		−6.81$\;\times 10^{-1}$	−6.81$\;\times 10^{-1}$	
Blood vessel malformation:slope	1.46$\;\times 10^{-2}$	1.46$\;\times 10^{-2}$		4.81$\;\times 10^{-2}$	4.81$\;\times 10^{-2}$	
SD[Blood vessel malformation:Intercept]	3.00	1.88		1.88	1.88	

*Note*. Blank entries occur when the parameter was not used in Scenario 2.

In Scenario 1, we keep the fixed effects parameters and the means of the random effects as they are for the PBC data in both groups, with the only difference being that the random‐effects variance–covariance matrix, D, is set to be the same in each group. In this setting, the differences between the groups are in the mean profiles and so we would expect the marginal prediction method to give the best prediction. In each group there is approximately the same amount of variability around the group average for each marker. The focus of this simulation scenario is on the marginal differences between groups.

In all the published comparisons of the three prediction approaches, either the random‐effects or marginal method has given the most accurate prediction. We are not aware of any studies in which the conditional method is the best. Further, we find it difficult to envisage a situation in which the conditional approach would outperform both marginal and random‐effects approach simultaneously. We suspect emphasising differences between the marginal profiles in each group would lead to the conditional approach outperforming the random‐effects method, but not the marginal method. In contrast, greater differences in the random‐effects structure would allow the conditional approach to outperform the marginal approach, but would be unlikely to lead to the conditional approach being better than the random‐effects approach.

Morrell et al. (2011) discuss the three approaches and speculate that the conditional approach may work well in the case where the residual error is large in comparison to the random‐effects variance. For our second scenario, we investigate further this possibility. Since only the continuous markers have a residual error term, in this scenario we only consider an MGLMM including the continuous markers in our simulation. In this case, the means and variances of the random effects are set to be the same in each group and the only difference is the value of the residual error. This reflects a scenario in which the measurement error in one group is larger than in the other group.

For each scenario, we simulated 100 data sets. The MGLMMs in each group were based on 10,000 iterations of 1:10 thinned MCMC after a burn in of 500 iterations. In each case, leave‐one‐out cross‐validation was used to provide individual patient predictions. MGLMMs were fitted using the GLMM_MCMC function, and LoDA was performed using the GLMM_longitDA2 function from the R package mixAK (Komárek & Komárková, 2014). The reported prediction accuracies and model parameters are based on the averages over 100 simulated data sets. Source code to reproduce the results is available from the corresponding author upon request.

### 4.1 Results for Scenario 1

Table 3 shows the mean parameter estimates for the MGLMM in each group across 100 simulated data sets. The simulated data sets approximate well the true model as shown by the low values of bias and MSE for most parameters. The coverage reports the proportion of times in which the true model parameter was within the estimated 95% credible interval for the parameter in the simulated data sets. The random slope variances for the continuous markers are poorly estimated in the simulated data sets. This is shown by the low coverage values of 0.45 and 0.76 in Group 0 and 0.57 and 0.50 in Group 1. We believe this may be due to the fact that the ‘true’ random‐effects variance for the slopes are smaller than the residual error making them difficult to estimate accurately (Table 2). However, the simulated data sets provide good approximations to the true GLMM parameters.

**Table 3:** Simulation study Scenario 1: Posterior means, highest posterior density (HPD) intervals, bias, standard deviation (SD), mean square error (MSE) and coverage for the fixed and random effects

	Group 0						Group 1					
	Posterior Mean	95% HPD Interval	SD	Bias	MSE	Coverage	Posterior Mean	95% HPD Interval	SD	Bias	MSE	Coverage
**Albumin**												
E[Albumin:Intercept]	3.69	(3.64,3.74)	4.03$\;\times 10^{-3}$	4.22$\;\times 10^{-3}$	6.35$\;\times 10^{-4}$	0.93	3.39	(3.29,3.49)	4.02$\;\times 10^{-3}$	−3.19$\;\times 10^{-3}$	2.08$\;\times 10^{-3}$	0.98
E[Albumin:slope]	−7.03$\;\times 10^{-3}$	(−9.35,−4.68)$\;\times 10^{-3}$	3.47$\;\times 10^{-4}$	−2.02$\;\times 10^{-4}$	2.15$\;\times 10^{-6}$	0.88	−1.42$\;\times 10^{-2}$	(−1.91,−0.93)$\;\times 10^{-2}$	2.76$\;\times 10^{-4}$	2.65$\;\times 10^{-4}$	6.85$\;\times 10^{-6}$	0.94
SD[Albumin:Intercept]	2.63$\;\times 10^{-1}$	(2.22,3.06)$\;\times 10^{-1}$	1.81$\;\times 10^{-3}$	−6.12$\;\times 10^{-4}$	4.10$\;\times 10^{-4}$	0.98	2.58$\;\times 10^{-1}$	(1.71,3.46)$\;\times 10^{-1}$	3.80$\;\times 10^{-3}$	−5.75$\;\times 10^{-3}$	2.77$\;\times 10^{-3}$	0.89
Corr[Albumin:Intercept,Albumin:slope]	2.09$\;\times 10^{-2}$	(−5.25,5.89)$\;\times 10^{-1}$	2.45$\;\times 10^{-2}$	8.55$\;\times 10^{-2}$	2.28$\;\times 10^{-2}$	1.00	3.96$\;\times 10^{-2}$	(−6.24,7.09)$\;\times 10^{-1}$	1.43$\;\times 10^{-2}$	1.04$\;\times 10^{-1}$	3.08$\;\times 10^{-2}$	0.99
Corr[Albumin:Intercept,log(Bilirubin):Intercept]	−1.75$\;\times 10^{-1}$	(−3.49,0.02)$\;\times 10^{-1}$	6.79$\;\times 10^{-3}$	2.21$\;\times 10^{-2}$	6.34$\;\times 10^{-3}$	0.99	−1.61$\;\times 10^{-1}$	(−4.97,1.81)$\;\times 10^{-1}$	1.14$\;\times 10^{-2}$	3.56$\;\times 10^{-2}$	2.87$\;\times 10^{-2}$	0.97
Corr[Albumin:Intercept,log(Bilirubin):slope]	1.73$\;\times 10^{-1}$	(−1.67,5.06)$\;\times 10^{-1}$	1.35$\;\times 10^{-2}$	−3.87$\;\times 10^{-2}$	2.44$\;\times 10^{-2}$	0.96	5.84$\;\times 10^{-2}$	(−5.94,6.94)$\;\times 10^{-1}$	1.38$\;\times 10^{-2}$	−1.53$\;\times 10^{-1}$	5.29$\;\times 10^{-2}$	0.98
Corr[Albumin:Intercept,Platelet:Intercept]	1.52$\;\times 10^{-1}$	(−0.24,3.27)$\;\times 10^{-1}$	8.84$\;\times 10^{-3}$	−3.88$\;\times 10^{-2}$	9.83$\;\times 10^{-3}$	0.93	1.12$\;\times 10^{-1}$	(−2.25,4.44)$\;\times 10^{-1}$	1.12$\;\times 10^{-2}$	−7.95$\;\times 10^{-2}$	3.13$\;\times 10^{-2}$	0.94
Corr[Albumin:Intercept,Platelet:slope]	6.59$\;\times 10^{-2}$	(−1.14,2.46)$\;\times 10^{-1}$	8.38$\;\times 10^{-3}$	−4.35$\;\times 10^{-2}$	9.15$\;\times 10^{-3}$	0.93	4.21$\;\times 10^{-2}$	(−3.00,3.86)$\;\times 10^{-1}$	1.17$\;\times 10^{-2}$	−6.73$\;\times 10^{-2}$	3.86$\;\times 10^{-2}$	0.91
Corr[Albumin:Intercept,Blood Vessel Malformations:Intercept]	−3.01$\;\times 10^{-1}$	(−5.22,‐0.76)$\;\times 10^{-1}$	9.16$\;\times 10^{-3}$	4.69$\;\times 10^{-2}$	1.36$\;\times 10^{-2}$	0.95	−2.58$\;\times 10^{-1}$	(−6.39,1.37)$\;\times 10^{-1}$	1.31$\;\times 10^{-2}$	9.01$\;\times 10^{-2}$	4.24$\;\times 10^{-2}$	0.96
SD[Albumin:slope]	3.47$\;\times 10^{-3}$	(0.65,7.13)$\;\times 10^{-3}$	6.27$\;\times 10^{-4}$	−4.30$\;\times 10^{-3}$	2.53$\;\times 10^{-5}$	0.45	3.98$\;\times 10^{-3}$	(0.36,9.15)$\;\times 10^{-3}$	3.65$\;\times 10^{-4}$	−3.79$\;\times 10^{-3}$	2.46$\;\times 10^{-5}$	0.57
Corr[Albumin:slope,log(Bilirubin):Intercept]	−2.06$\;\times 10^{-2}$	(−5.58,5.18)$\;\times 10^{-1}$	2.22$\;\times 10^{-2}$	−2.22$\;\times 10^{-2}$	1.29$\;\times 10^{-2}$	0.99	−1.31$\;\times 10^{-2}$	(−6.54,6.34)$\;\times 10^{-1}$	1.23$\;\times 10^{-2}$	−1.47$\;\times 10^{-2}$	1.74$\;\times 10^{-2}$	0.99
Corr[Albumin:slope,log(Bilirubin):slope]	−2.65$\;\times 10^{-2}$	(−6.18,5.74)$\;\times 10^{-1}$	2.55$\;\times 10^{-2}$	2.06$\;\times 10^{-1}$	6.09$\;\times 10^{-2}$	0.99	‐1.03$\;\times 10^{-2}$	(−7.62,7.46)$\;\times 10^{-1}$	1.14$\;\times 10^{-2}$	2.23$\;\times 10^{-1}$	5.82$\;\times 10^{-2}$	1.00
Corr[Albumin:slope,Platelet:Intercept]	−1.69$\;\times 10^{-1}$	(−6.92,3.87)$\;\times 10^{-1}$	3.54$\;\times 10^{-2}$	8.78$\;\times 10^{-2}$	2.56$\;\times 10^{-2}$	1.00	−5.97$\;\times 10^{-2}$	(−6.86,5.84)$\;\times 10^{-1}$	1.30$\;\times 10^{-2}$	1.97$\;\times 10^{-1}$	5.69$\;\times 10^{-2}$	0.99
Corr[Albumin:slope,Platelet:slope]	−1.54$\;\times 10^{-1}$	(−6.54,3.73)$\;\times 10^{-1}$	3.06$\;\times 10^{-2}$	1.07$\;\times 10^{-1}$	3.29$\;\times 10^{-2}$	0.99	−8.70$\;\times 10^{-2}$	(−6.94,5.46)$\;\times 10^{-1}$	1.39$\;\times 10^{-2}$	1.73$\;\times 10^{-1}$	5.08$\;\times 10^{-2}$	0.98
Corr[Albumin:slope,Blood Vessel Malformations:Intercept]	1.01$\;\times 10^{-1}$	(−4.86,6.62)$\;\times 10^{-1}$	3.09$\;\times 10^{-2}$	−1.26$\;\times 10^{-1}$	3.14$\;\times 10^{-2}$	0.98	2.11$\;\times 10^{-2}$	(−6.62,6.96)$\;\times 10^{-1}$	1.49$\;\times 10^{-2}$	−2.06$\;\times 10^{-1}$	6.47$\;\times 10^{-2}$	0.99
**log(Bilirubin)**												
E[log(Bilirubin):Intercept]	1.67$\;\times 10^{-2}$	(−1.06,1.39)$\;\times 10^{-1}$	4.04$\;\times 10^{-3}$	−4.57$\;\times 10^{-3}$	3.71$\;\times 10^{-3}$	0.96	1.24	(0.99,1.49)	8.51$\;\times 10^{-3}$	9.60$\;\times 10^{-3}$	1.62$\;\times 10^{-2}$	0.96
E[log(Bilirubin):slope]	1.00$\;\times 10^{-2}$	(0.69,1.32)$\;\times 10^{-2}$	1.27$\;\times 10^{-4}$	8.22$\;\times 10^{-5}$	3.41$\;\times 10^{-6}$	0.91	2.31$\;\times 10^{-2}$	(1.65,2.99)$\;\times 10^{-2}$	3.44$\;\times 10^{-4}$	−6.91$\;\times 10^{-4}$	1.33$\;\times 10^{-5}$	0.94
SD[log(Bilirubin):Intercept]	8.42$\;\times 10^{-1}$	(7.52,9.35)$\;\times 10^{-1}$	3.03$\;\times 10^{-3}$	−3.32$\;\times 10^{-3}$	2.37$\;\times 10^{-3}$	0.97	8.38$\;\times 10^{-1}$	(0.66,1.03)	6.08$\;\times 10^{-3}$	−7.28$\;\times 10^{-3}$	7.98$\;\times 10^{-3}$	0.96
Corr[log(Bilirubin):Intercept,log(Bilirubin):slope]	−1.33$\;\times 10^{-1}$	(−4.25,1.78)$\;\times 10^{-1}$	1.11$\;\times 10^{-2}$	4.12$\;\times 10^{-2}$	2.26$\;\times 10^{-2}$	0.93	−4.34$\;\times 10^{-2}$	(−6.45,5.95)$\;\times 10^{-1}$	1.31$\;\times 10^{-2}$	1.31$\;\times 10^{-1}$	4.00$\;\times 10^{-2}$	0.99
Corr[log(Bilirubin):Intercept,Platelet:Intercept]	2.35$\;\times 10^{-1}$	(0.97,3.70)$\;\times 10^{-1}$	4.56$\;\times 10^{-3}$	−1.25$\;\times 10^{-2}$	4.23$\;\times 10^{-3}$	0.98	2.12$\;\times 10^{-1}$	(−0.60,4.78)$\;\times 10^{-1}$	8.66$\;\times 10^{-3}$	−3.48$\;\times 10^{-2}$	1.67$\;\times 10^{-2}$	0.96
Corr[log(Bilirubin):Intercept,Platelet:slope]	−1.86$\;\times 10^{-1}$	(−3.28,−0.42)$\;\times 10^{-1}$	4.66$\;\times 10^{-3}$	9.56$\;\times 10^{-4}$	5.38$\;\times 10^{-3}$	0.93	−1.49$\;\times 10^{-1}$	(−4.27,1.34)$\;\times 10^{-1}$	9.22$\;\times 10^{-3}$	3.77$\;\times 10^{-2}$	2.06$\;\times 10^{-2}$	0.94
Corr[log(Bilirubin):Intercept,Blood Vessel Malformations:Intercept]	2.73$\;\times 10^{-1}$	(0.82,4.60)$\;\times 10^{-1}$	6.34$\;\times 10^{-3}$	2.29$\;\times 10^{-3}$	8.31$\;\times 10^{-3}$	0.96	2.48$\;\times 10^{-1}$	(−0.88,5.74)$\;\times 10^{-1}$	1.12$\;\times 10^{-2}$	−2.19$\;\times 10^{-2}$	2.70$\;\times 10^{-2}$	0.95
SD[log(Bilirubin):slope]	1.20$\;\times 10^{-2}$	(0.64,1.72)$\;\times 10^{-2}$	4.52$\;\times 10^{-4}$	−2.93$\;\times 10^{-3}$	2.17$\;\times 10^{-5}$	0.76	7.03$\;\times 10^{-3}$	(0.05,1.55)$\;\times 10^{-2}$	4.76$\;\times 10^{-4}$	−7.90$\;\times 10^{-3}$	8.64$\;\times 10^{-5}$	0.50
Corr[log(Bilirubin):slope,Platelet:Intercept]	−1.75$\;\times 10^{-1}$	(−4.77,1.28)$\;\times 10^{-1}$	1.12$\;\times 10^{-2}$	−5.37$\;\times 10^{-3}$	1.87$\;\times 10^{-2}$	0.94	−6.26$\;\times 10^{-2}$	(−6.68,5.62)$\;\times 10^{-1}$	1.30$\;\times 10^{-2}$	1.07$\;\times 10^{-1}$	3.67$\;\times 10^{-2}$	0.99
Corr[log(Bilirubin):slope,Platelet:slope]	1.10$\;\times 10^{-1}$	(−1.88,4.07)$\;\times 10^{-1}$	1.01$\;\times 10^{-2}$	−1.48$\;\times 10^{-2}$	2.24$\;\times 10^{-2}$	0.94	6.57$\;\times 10^{-2}$	(−5.44,6.61)$\;\times 10^{-1}$	1.26$\;\times 10^{-2}$	−5.91$\;\times 10^{-2}$	2.35$\;\times 10^{-2}$	1.00
Corr[log(Bilirubin):slope,Blood Vessel Malformations:Intercept]	1.92$\;\times 10^{-2}$	(−3.55,3.97)$\;\times 10^{-1}$	1.35$\;\times 10^{-2}$	1.11$\;\times 10^{-2}$	3.06$\;\times 10^{-2}$	0.95	1.62$\;\times 10^{-2}$	(−6.44,6.77)$\;\times 10^{-1}$	1.41$\;\times 10^{-2}$	8.09$\;\times 10^{-3}$	2.87$\;\times 10^{-2}$	0.99
**Platelet count**												
E[Platelet:Intercept]	5.54	(5.49,5.59)	1.58$\;\times 10^{-3}$	2.20$\;\times 10^{-3}$	5.03$\;\times 10^{-4}$	0.96	5.46	(5.36,5.55)	3.12$\;\times 10^{-3}$	−4.86$\;\times 10^{-3}$	1.70$\;\times 10^{-3}$	0.98
E[Platelet:slope]	−4.29$\;\times 10^{-3}$	(−6.45,−2.14)$\;\times 10^{-3}$	7.02$\;\times 10^{-5}$	4.70$\;\times 10^{-6}$	1.25$\;\times 10^{-6}$	0.94	−1.14$\;\times 10^{-2}$	(−1.59,‐0.70)$\;\times 10^{-2}$	1.42$\;\times 10^{-4}$	−3.02$\;\times 10^{-5}$	4.33$\;\times 10^{-6}$	0.95
SD[Platelet:Intercept]	3.49$\;\times 10^{-1}$	(3.15,3.85)$\;\times 10^{-1}$	1.13$\;\times 10^{-3}$	4.27$\;\times 10^{-3}$	3.38$\;\times 10^{-4}$	0.96	3.49$\;\times 10^{-1}$	(2.81,4.23)$\;\times 10^{-1}$	2.34$\;\times 10^{-3}$	4.34$\;\times 10^{-3}$	1.39$\;\times 10^{-3}$	0.94
Corr[Platelet:Intercept,Platelet:slope]	6.66$\;\times 10^{-2}$	(−0.75,2.08)$\;\times 10^{-1}$	4.65$\;\times 10^{-3}$	5.20$\;\times 10^{-3}$	5.77$\;\times 10^{-3}$	0.93	6.77$\;\times 10^{-2}$	(−2.11,3.45)$\;\times 10^{-1}$	9.28$\;\times 10^{-3}$	6.25$\;\times 10^{-3}$	1.59$\;\times 10^{-2}$	0.97
Corr[Platelet:Intercept,Blood vessel malformations:Intercept]	−2.57$\;\times 10^{-1}$	(−4.40,−0.71)$\;\times 10^{-1}$	6.11$\;\times 10^{-3}$	−9.29$\;\times 10^{-3}$	9.27$\;\times 10^{-3}$	0.94	−2.23$\;\times 10^{-1}$	(−5.42,1.05)$\;\times 10^{-1}$	1.04$\;\times 10^{-2}$	2.47$\;\times 10^{-2}$	2.60$\;\times 10^{-2}$	0.97
SD[Platelet:slope]	1.52$\;\times 10^{-2}$	(1.37,1.69)$\;\times 10^{-2}$	5.20$\;\times 10^{-5}$	9.68$\;\times 10^{-5}$	6.37$\;\times 10^{-7}$	0.95	1.55$\;\times 10^{-2}$	(1.23,1.90)$\;\times 10^{-2}$	1.09$\;\times 10^{-4}$	3.74$\;\times 10^{-4}$	2.92$\;\times 10^{-6}$	0.97
Corr[Platelet:slope,Blood vessel malformations:Intercept]	−7.64$\;\times 10^{-2}$	(−2.72,1.20)$\;\times 10^{-1}$	6.45$\;\times 10^{-3}$	3.96$\;\times 10^{-3}$	9.90$\;\times 10^{-3}$	0.96	−7.61$\;\times 10^{-2}$	(−4.16,2.68)$\;\times 10^{-1}$	1.13$\;\times 10^{-2}$	4.24$\;\times 10^{-3}$	2.39$\;\times 10^{-2}$	0.97
**Blood vessel malformations**												
E[Blood vessel malformations:Intercept]	‐2.55	(−3.12,−2.00)	1.93$\;\times 10^{-2}$	−7.35$\;\times 10^{-3}$	1.19$\;\times 10^{-1}$	0.89	−6.74$\;\times 10^{-1}$	(−1.48,0.11)	2.57$\;\times 10^{-2}$	6.45$\;\times 10^{-3}$	1.82$\;\times 10^{-1}$	0.92
Blood vessel malformations:Slope	1.37$\;\times 10^{-2}$	(−1.03,3.78)$\;\times 10^{-2}$	7.98$\;\times 10^{-4}$	−8.53$\;\times 10^{-4}$	1.84$\;\times 10^{-4}$	0.88	4.63$\;\times 10^{-2}$	(0.67,8.66)$\;\times 10^{-2}$	1.35$\;\times 10^{-3}$	−1.77$\;\times 10^{-3}$	3.96$\;\times 10^{-4}$	0.95
SD[Blood vessel malformations:Intercept]	1.89	(1.39,2.40)	1.84$\;\times 10^{-2}$	4.76$\;\times 10^{-3}$	1.02$\;\times 10^{-1}$	0.89	1.82	(1.02,2.69)	3.42$\;\times 10^{-2}$	−5.73$\;\times 10^{-2}$	2.71$\;\times 10^{-1}$	0.91

*Note*. These measurements are the average of 100 simulations.

**Figure 3:**
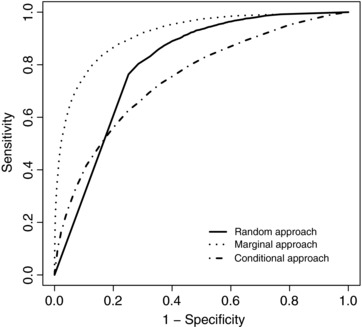
Receiver operating characteristic curves of the LoDA using the random‐effects (solid), marginal (dotted) and conditional (dot‐dashed) prediction methods for Scenario 1

Under Scenario 1, the marginal method gave the best predictive accuracy in terms of AUC, specificity, PCC and PPV (Table 4). The choice of method is not so clear‐cut as in Table 1 as the random‐effects approach gives the best sensitivity and NPV, although with a much worse specificity. These accuracies were calculated by selecting the optimal cut‐off for each simulated data set and averaging the respective sensitivities, specificities, etc. Nevertheless Figure 3, which averages the sensitivity and specificity at each cut‐off across the 100 simulated data sets, shows that the marginal approach consistently outperforms the other methods. This is consistent with what we expected since the main differences between the groups were in the fixed effects and expected values of the random effects.

**Table 4:** Scenario 1 prediction accuracy from leave‐one‐out cross‐validation of random‐effects, marginal and conditional prediction; the reported values are the averages over the 100 simulated data sets

	Random	Marginal	Conditional
Cut‐off	0.81	0.19	0.12
Sensitivity	0.93	0.85	0.70
Specificity	0.71	0.85	0.73
PCC	0.75	0.85	0.73
AUC	0.84	0.91	0.76
PPV	0.57	0.59	0.40
NPV	0.98	0.96	0.96

We conclude from Scenario 1 that when the main differences between the groups are in the mean longitudinal evolution, the marginal method will be the best tool to classify patients. This effect was true in the case of Brant et al. (2003) and Morrell et al. (2011), where the marginal approach was shown to give the best classification results. The expected PSA level was seen to increase substantially between visits for patients who developed prostate cancer, and so the marginal approach was able to detect a difference between the largely stable PSA profile of healthy patients and the generally increasing longitudinal PSA profiles of patients who would ultimately develop prostate cancer. By contrast, Figure 1 and Tables 2 and 3 of Komárek et al. (2010), in which the random‐effects approach gave best prediction, show that although there were some differences between the mean longitudinal profiles of each group there were also substantial differences in the patient‐specific variability around the group mean in each group. Incorporating this additional information (which the random‐effects approach does) led to the random‐effects approach most accurately identifying patients who would require liver transplant or die.

### 4.2 Results for Scenario 2

Table 6 shows that the bias, standard deviation and MSE of the estimated parameters was generally very low demonstrating that each simulated sample approximated the true model well.

**Figure 4:**
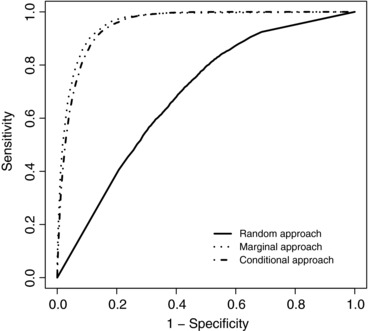
Receiver operating characteristic curves of the LoDA using the random‐effects (solid), marginal (dotted) and conditional (dot‐dashed) prediction methods for Scenario 2

In Scenario 2, the only difference between the two groups is the value of the residual variance (Table 2). The random‐effects approach is unable to detect this difference. In addition, since the residual variance is larger than the random‐effects variances, the model is unable to make accurate estimates of the individual random effects leading to poor prediction (Table 5 and Fig. 4). The poor estimation of the random‐effects parameters is also seen in the worse coverage rates in Table 6. The marginal and conditional approaches are still able to make accurate classification of patients with 90% and 89% of patients correctly identified, respectively. It is noticeable however, that even in a situation which we thought would most favour the conditional approach the marginal approach is just as good on all measures of accuracy. Figure 4 shows that whilst the marginal and conditional approaches classify the patients well, the random‐effects approach performs little better than chance.

**Table 5:** Scenario 2 prediction accuracy from leave‐one‐out cross‐validation of random‐effects, marginal and conditional prediction; the reported values are the averages over the 100 simulated data sets

	Random	Marginal	Conditional
Cut‐off	0.56	0.26	0.58
Sensitivity	0.78	0.92	0.92
Specificity	0.70	0.89	0.88
PCC	0.72	0.90	0.89
AUC	0.74	0.96	0.95
PPV	0.55	0.69	0.66
NPV	0.90	0.98	0.93

**Table 6:** Simulation study Scenario 2: Posterior means, highest posterior density (HPD) intervals, bias, standard deviation (SD), mean square error (MSE) and coverage for the fixed and random effects

	Group 0						Group 1					
	Posterior Mean	95% HPD Interval	SD	Bias	MSE	Coverage	Posterior Mean	95% HPD Interval	SD	Bias	MSE	Coverage
**Albumin**												
E[Albumin:Intercept]	3.00	(2.97,3.03)	2.80$\;\times 10^{-3}$	−2.04$\;\times 10^{-3}$	3.73$\;\times 10^{-4}$	0.89	3.00	(2.96,3.04)	1.89$\;\times 10^{-3}$	3.45$\;\times 10^{-3}$	4.00$\;\times 10^{-4}$	0.92
E[Albumin:slope]	−9.63$\times 10^{-5}$	(−2.37,2.10)$\;\times 10^{-3}$	2.12$\;\times 10^{-4}$	−9.63$\;\times 10^{-5}$	1.49$\;\times 10^{-6}$	0.92	−3.08$\;\times 10^{-4}$	(−3.01,2.38)$\;\times 10^{-3}$	1.31$\;\times 10^{-4}$	−3.08$\;\times 10^{-4}$	1.88$\;\times 10^{-6}$	0.92
SD[Albumin:Intercept]	4.19$\;\times 10^{-2}$	(0.34,9.07)$\;\times 10^{-2}$	4.21$\;\times 10^{-3}$	−2.31$\;\times 10^{-2}$	1.06$\;\times 10^{-3}$	0.84	4.28$\;\times 10^{-2}$	(0.68,8.68)$\;\times 10^{-2}$	2.10$\;\times 10^{-3}$	−2.22$\;\times 10^{-2}$	1.19$\;\times 10^{-3}$	0.76
Corr[Albumin:Intercept,Albumin:slope]	−7.48$\;\times 10^{-3}$	(−8.53,8.51)$\;\times 10^{-1}$	2.02$\;\times 10^{-2}$	5.72$\;\times 10^{-2}$	1.26$\;\times 10^{-2}$	1.00	2.61$\;\times 10^{-2}$	(−8.02,8.50)$\;\times 10^{-1}$	1.18$\;\times 10^{-2}$	9.07$\;\times 10^{-2}$	2.60$\;\times 10^{-2}$	1.00
Corr[Albumin:Intercept,log(Bilirubin):Intercept]	1.50$\;\times 10^{-2}$	(−8.38,8.64)$\;\times 10^{-1}$	2.32$\;\times 10^{-2}$	2.12$\;\times 10^{-1}$	5.79$\;\times 10^{-2}$	1.00	3.21$\;\times 10^{-3}$	(−8.46,8.64)$\;\times 10^{-1}$	1.10$\;\times 10^{-2}$	2.00$\;\times 10^{-1}$	5.28$\;\times 10^{-2}$	1.00
Corr[Albumin:Intercept,log(Bilirubin):slope]	3.21$\;\times 10^{-2}$	(−7.43,7.95)$\;\times 10^{-1}$	2.46$\;\times 10^{-2}$	−1.79$\;\times 10^{-1}$	7.77$\;\times 10^{-2}$	0.97	1.01$\;\times 10^{-1}$	(−6.29,7.96)$\;\times 10^{-1}$	1.57$\;\times 10^{-2}$	−1.10$\;\times 10^{-1}$	5.22$\;\times 10^{-2}$	0.98
SD[Albumin:slope]	2.13$\;\times 10^{-3}$	(0.07,5.38)$\;\times 10^{-3}$	2.54$\;\times 10^{-4}$	−5.63$\;\times 10^{-3}$	3.38$\;\times 10^{-5}$	0.16	2.91$\;\times 10^{-3}$	(0.56,5.79)$\;\times 10^{-3}$	1.32$\;\times 10^{-4}$	−4.85$\;\times 10^{-3}$	2.75$\;\times 10^{-5}$	0.24
Corr[Albumin:slope,log(Bilirubin):Intercept]	1.18$\;\times 10^{-2}$	(−8.59,8.73)$\;\times 10^{-1}$	2.35$\;\times 10^{-2}$	1.02$\;\times 10^{-2}$	8.28$\;\times 10^{-3}$	1.00	−2.69$\;\times 10^{-2}$	(−8.80,8.37)$\;\times 10^{-1}$	1.06$\;\times 10^{-2}$	−2.84$\;\times 10^{-2}$	8.65$\;\times 10^{-3}$	1.00
Corr[Albumin:slope,log(Bilirubin):slope]	−4.95$\;\times 10^{-2}$	(−8.46,7.84)$\;\times 10^{-1}$	2.98$\;\times 10^{-2}$	1.83$\;\times 10^{-1}$	6.26$\;\times 10^{-2}$	0.99	−8.38$\;\times 10^{-2}$	(−7.91,6.32)$\;\times 10^{-1}$	1.50$\;\times 10^{-2}$	1.49$\;\times 10^{-1}$	6.69$\;\times 10^{-2}$	0.98
**log(Bilirubin)**												
E[log(Bilirubin):Intercept]	1.00	(0.96,1.04)	3.05$\;\times 10^{-3}$	−3.92$\;\times 10^{-4}$	3.58$\;\times 10^{-4}$	0.96	1.00	(0.96,1.04)	1.74$\;\times 10^{-3}$	7.55$\;\times 10^{-4}$	3.71$\;\times 10^{-4}$	0.92
E[log(Bilirubin):slope]	8.35$\;\times 10^{-5}$	(−3.20,3.37)$\;\times 10^{-3}$	1.95$\;\times 10^{-4}$	8.35$\;\times 10^{-5}$	2.18$\;\times 10^{-6}$	0.98	3.08$\;\times 10^{-4}$	(−3.69,4.28)$\;\times 10^{-3}$	1.50$\;\times 10^{-4}$	3.08$\;\times 10^{-4}$	4.18$\;\times 10^{-6}$	0.93
SD[log(Bilirubin):Intercept]	3.35$\;\times 10^{-2}$	(0.03,9.02)$\;\times 10^{-2}$	3.39$\;\times 10^{-3}$	2.23$\;\times 10^{-2}$	1.11$\;\times 10^{-3}$	0.99	1.79$\;\times 10^{-2}$	(0.00,5.29)$\;\times 10^{-2}$	1.36$\;\times 10^{-3}$	6.65$\;\times 10^{-3}$	2.21$\;\times 10^{-4}$	0.98
Corr[log(Bilirubin):Intercept,log(Bilirubin):slope]	−1.11$\;\times 10^{-2}$	(−8.24,8.29)$\;\times 10^{-1}$	2.61$\;\times 10^{-2}$	1.64$\;\times 10^{-1}$	5.96$\;\times 10^{-2}$	1.00	−1.66$\;\times 10^{-2}$	(−8.36,8.30)$\;\times 10^{-1}$	1.48$\;\times 10^{-2}$	1.58$\;\times 10^{-1}$	4.98$\;\times 10^{-2}$	0.99
SD[log(Bilirubin):slope]	1.02$\;\times 10^{-2}$	(0.59,1.43)$\;\times 10^{-2}$	2.67$\;\times 10^{-4}$	−4.74$\;\times 10^{-3}$	2.95$\;\times 10^{-5}$	0.38	1.10$\;\times 10^{-2}$	(0.79,1.43)$\;\times 10^{-2}$	1.06$\;\times 10^{-4}$	−3.93$\;\times 10^{-3}$	1.82$\;\times 10^{-5}$	0.39

*Note*. These measurements are the average of 100 simulations.

According to Sections 4.1 and 4.2 of Komárek et al. (2010), in the case of continuous longitudinal markers, the normal distributions used to calculate group membership probabilities for both conditional and marginal methods use the residual error. For the marginal approach the variance of the multivariate normal distribution is influenced by the residual variance whilst for the conditional approach both mean and variance are affected. The normal distribution for the random‐effects approach does not use the residual variance and relies on an estimate of the individual random effects, which we noted above, has been poorly estimated due to the high residual error. This demonstrates why both conditional and marginal methods are able to detect a difference in the residual variance between the groups but the random‐effects approach cannot.

**Figure 5:**
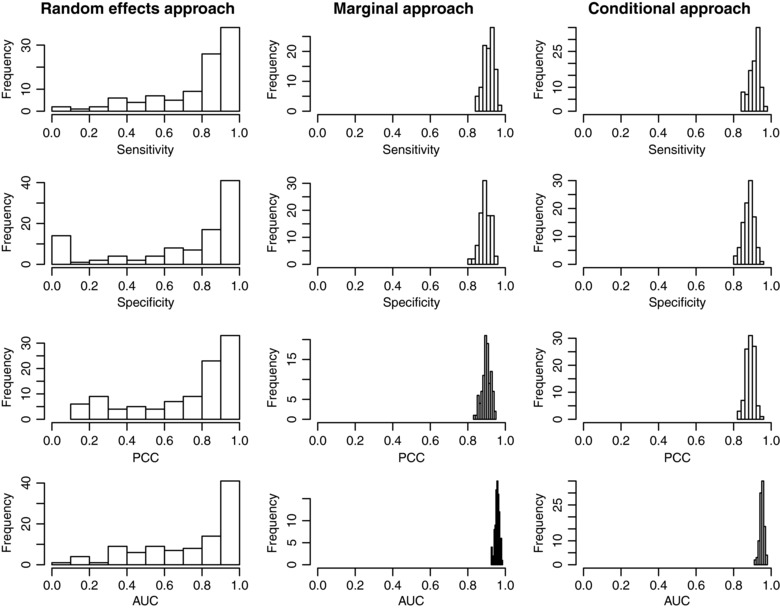
Histograms showing the sensitivity, specificity, PCC and AUC of each of the three approaches for each of the 100 simulated data sets under Scenario 2

It should be noted that we observed large variation in the prediction accuracy of the random‐effects approach over each simulated data sets. This accounts for the fact that the average ‘best’ sensitivities and specificities in Table 5 are noticeably better than the ROC curve for the random‐effects approach in Figure 4 (where sensitivity and specificity are averaged across the 100 data sets at each cut‐off). The prediction accuracy of the marginal and conditional approaches were, in contrast, much more stable. This is demonstrated in Figure 5, which shows the sensitivity, specificity, PCC and AUC for each simulated data set under Scenario 2. For each of the measures considered, the values in each simulated data set are very similar for both marginal and conditional methods. However, the inability of the random‐effects approach to correctly estimate the individual patient random effects leads to very unstable estimates of sensitivity and specificity for example (a similar effect was observed in Scenario 1). It is noticeable that many of the simulated data sets gave sensitivity of 1 and specificity of 0, reflecting the fact that the random‐effects approach was unable to distinguish between the two groups. This leads us to conclude that when analysing data in which there seems to be a high likelihood of large measurement error, researchers should be wary about using the random‐effects approach and may wish to focus on the marginal approach.

## 5 DISCUSSION

In this paper, we have compared three approaches to predicting group membership using LoDA, specifically the marginal, conditional and random‐effects approaches. These approaches have been compared previously using a number of real data sets with contrasting results regarding which approach gives the most accurate prediction. The marginal and random‐effects approaches are shown to give the most accurate classification in an application of multivariate LoDA to the real data of the Mayo PBC study. We explored the three approaches further by way of a simulation study in which we explored two scenarios designed to favour the marginal and conditional approaches.

When the average profile is noticeably different between prognostic groups then the marginal approach is expected to provide good classification accuracy. However, if the main difference between prognostic groups is dominated by the variability about the mean profile (differences in subject‐specific variability across the groups) then the marginal approach is not able to distinguish patients as well and the random‐effects approach is expected to work best.

The 95% credible interval coverage for the simulations indicated that for some of the parameters the coverage was considerably below 95%, suggesting poor estimation. On the other hand, a coverage around 99% was observed for some of the random‐effects covariance terms, which may have been influenced by (i) the magnitude of the true values, which tend to be small in comparison to the residual error variance and (ii) the fact that we are attempting to fit a reasonably complicated model to fairly small numbers of patients (200 and 50 for Group 0 and Group 1, respectively), and with only four observations per patient. It is possible that over a larger number of simulated data sets, or with more repeated measurements of each marker, more precise credible intervals could be calculated which would in turn influence the coverage.

Although three approaches have been reported in the literature (and compared in this paper), we have been unable to simulate a scenario in which the conditional approach works better than the marginal and random‐effects approaches simultaneously. The conditional approach seems to offer little additional value to these two approaches.

There has been insufficient guidance as to which prediction approach to use in applications of LoDA. We suggest that a data analyst first plots longitudinal profiles of their markers for patients in each prognostic group. If there are differences in the group mean profiles and similar between‐ and within‐subject variability between groups, then the marginal approach should be expected to provide the best accuracy results.

If, in addition there seems to be a difference in the level of variability about the group mean in each group, then the random‐effects approach is expected to offer additional information leading to more accurate classification. However, if the variability between patients is dominated by a large measurement error, then the random‐effects approach should be avoided since estimates of the individual random effects are inaccurate. In such a case, the marginal approach would be preferred. In addition, if there are only a few repeated measurements per patient, it may be that estimates of individual patient random effects are not sufficiently precise to detect differences between the groups. In the case of only a few measurements per patient, we suggest the marginal approach is a good first option.

Further work could consider the effect that group prevalence has on the prediction accuracy of each method. The overall sample size and the number of longitudinal observations per patient may also influence the choice of which approach is preferable (e.g. the random‐effects approach relies on having enough data collected to properly characterise subject‐specific profiles).

## CONFLICT OF INTEREST

The authors have declared no conflict of interest.
